# Nervous decision-making: to divide or differentiate

**DOI:** 10.1016/j.tig.2014.04.001

**Published:** 2014-06

**Authors:** Laura J.A. Hardwick, Anna Philpott

**Affiliations:** Department of Oncology, University of Cambridge, Hutchison/MRC Research Centre, Cambridge Biomedical Campus, Cambridge, CB2 0XZ, UK

**Keywords:** cell cycle, differentiation, neurogenesis

## Abstract

The intricate balance between proliferation and differentiation is of fundamental importance in the development of the central nervous system (CNS). The division versus differentiation decision influences both the number and identity of daughter cells produced, thus critically shaping the overall microstructure and function of the CNS. During the past decade, significant advances have been made to characterise the changes in the cell cycle during differentiation, and to uncover the multiple bidirectional links that coordinate these two processes. Here, we explore the nature and mechanistic basis of these links in the context of the developing CNS, highlighting new insights into transcriptional, post-translational, and epigenetic levels of interaction.

## Highlights

• Multiple mechanisms coordinate the cell cycle and neuronal differentiation.
• Lengthening of G1 phase is functionally important for differentiation.
• Cell cycle components can directly and independently affect neurogenesis.
• Differentiation factors can directly affect the cell cycle structure and machinery.

## Neurogenesis and the cell cycle

Formation of the CNS requires exquisite regulation of precursor proliferation, cell cycle exit, and differentiation to generate the diverse array of neurons and glial cells at the correct time and place. During neurogenesis, the population of precursor cells can undergo three different modes of division (see Glossary): early proliferative divisions are critical for expanding the precursor pool, and the timing of the switch to asymmetric and later symmetric neurogenic divisions ultimately determines differential rates of growth in different regions of the nervous system and, thus, the overall microstructure and function [1]. Neurogenesis follows a temporal pattern, with precursor cells changing their competence and forming different cell types over time [2]; therefore, maintenance of the precursor pool is essential to enable the full repertoire of cell types to form [3]. Furthermore, this highly regulated temporal production of different cell types is conserved throughout amniote evolution [4], but modifications to progenitor cell number, location, and proliferative capacity has enabled expansion of the mammalian cortex and the emergence of gyrencephaly that characterises the primate brain [5,6]. Indeed, cell fate specification throughout embryogenesis is intimately linked with the cell cycle. For example, early lineage determination of proliferating pluripotent stem cells occurs in different phases of the cell cycle, with endodermal versus neuroectodermal specification occurring in early or late G1 phase, respectively [7]. Similarly, the characteristic six-layered architecture of the mammalian cortex is formed by sequential waves of neurogenesis and newborn neurons migrating radially to the cortical plate, with terminal laminar fate determined during the final S or G2 phase of the proliferating precursors [8].

**Figure 1:**
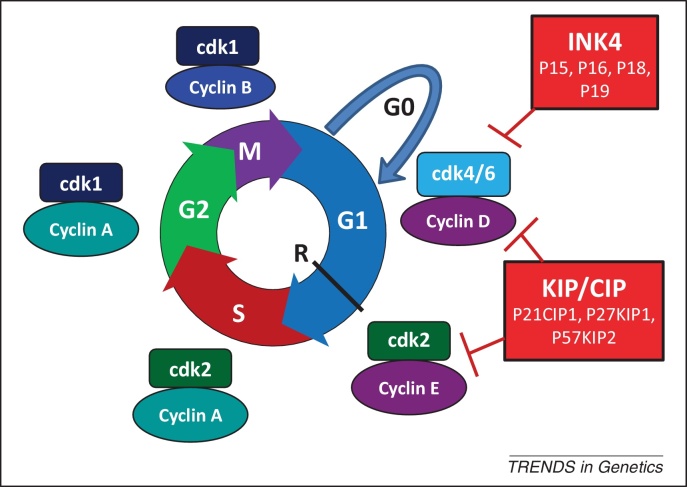
The eukaryotic cell cycle. The eukaryotic cell cycle comprises four sequential phases. Interphase is the collective term for the two gap phases (G1 and G2), during which cell growth occurs, and the intervening S phase when nuclear DNA is replicated. M phase (mitosis) constitutes nuclear division and cytokinesis. G1 provides the time in which the cell is responsive to extrinsic signals that influence the decision to either withdraw from the cell cycle into the quiescent G0 phase, or to pass the restriction point (R) and become committed to a further round of cell division. Checkpoints occur during the cell cycle to ensure successful completion of key events, such as DNA replication and chromosome alignment, before the cell passes into the next respective phase. Complex regulation of the transcription, post-translational modification, and protein degradation of key components ensures a unidirectional passage. Transition between phases is driven by specific combinations of cyclin-dependent kinases (cdks) with their respective activating cyclin partners, shown in the diagram adjacent to their approximate position in the cell cycle. For example, during the G1 phase, cyclin-D-cdk4/6 phosphorylates and inhibits the retinoblastoma-associated protein (Rb), thus releasing the inhibition on the E2F transcription factors and leading to expression of the genes necessary for cell cycle progression into S phase. The overall rate of cell cycle progression is determined by the relative activity of the activating cyclin-cdk complexes and the inhibitory proteins of the INK4 family that inhibit cdk4 and cdk6 in G1 phase, and the KIP/CIP family that has more widespread inhibitory action through the cell cycle (reviewed in [53]).

The coordination between the events of the cell cycle (Figure 1) and the changing modes of precursor cell division has been largely unexplored until relatively recently. Surprisingly, despite the intimate relation between the cell cycle and differentiation, these processes can be experimentally uncoupled, and cell cycle exit is neither a prerequisite for neurogenesis [9], nor always a consequence of neuronal differentiation [10]. Nevertheless, recent advances have characterised the cell cycle dynamics, transcriptome, and proteome accompanying the transition from proliferating precursor cell to differentiating neuron, uncovering the existence of multiple links between components of the cell cycle and differentiation machinery. Here, we focus on exploring these links and their underlying mechanistic basis in the context of the developing CNS.

## Cell cycle length: switching the balance from proliferation to differentiation

### Changes in cell cycle dynamics during differentiation

Recent findings indicate that the duration of G1 and S phase may have a crucial role in the precursor maintenance versus differentiation decision, which has been widely studied in the mouse CNS. Early studies in mouse ventricular zone (VZ) precursor cells characterised the progressive lengthening of the cell cycle during the neurogenic period, from 8 h at embryonic day (E)11 up to 18 h by E16, due to a lengthening of the G1 phase from 3 to 12 h [11], but this did not distinguish between precursors undergoing different modes of cell division. More recently, the Tis21-GFP knock-in reporter mouse has been used to express GFP selectively in the precursor cells undergoing neurogenic but not proliferative divisions [12], and subsequent work has used molecular markers (Pax6 and Tbr2) to further differentiate the apical progenitor (AP) and basal progenitor (BP) populations [13].

Proliferating precursor cells display a 3.3-fold longer S phase than their neurogenic counterparts, possibly due to a greater investment in fidelity of DNA replication [13] and similar changes in S phase duration have been reported following experimental manipulation to promote proliferative divisions of precursors [14]. G1 lengthening is associated with the switch to neuron-generating cell fate [12], specifically during the transition from AP to BP [13].

More recent advances have been made using imaging techniques to analyse cell cycle dynamics in live stem cell cultures, with several groups utilising the fluorescence ubiquitination cell cycle indicator (FUCCI) reporter system [15] to label live cells in different phases of the cell cycle. These studies demonstrate clear links between cell cycle parameters and the propensity to differentiate. Pluripotency in mouse embryonic stem cells (mESC) is associated with a short G1 phase of approximately 2 h within a cell cycle of approximately 14 h, and cells with faster cell cycles express lower levels of differentiation markers [16]. Furthermore, pluripotency can be promoted in culture by stimulation of the LIF signalling path, and this may partly be due to an accelerated transit through G1 [17]. Induction of differentiation results in a doubling of G1 length [16,17] with similar results reported in human ESCs [18].

### The cell cycle length hypothesis: the importance of G1

The functional link between G1 length and the decision to proliferate or differentiate has led to ‘The cell cycle length hypothesis’, based on a model whereby the length of the G1 phase determines whether a fate-determining signal will have sufficient time to produce an effect [19]. This paradigm is repeatedly seen across multiple different stem cell lineages [20] and recent work has demonstrated that G1-phase ESCs have an increased susceptibility to differentiate when compared with equivalent S or G2 phase cells [17,21].

The past decade has seen the development of multiple different experimental approaches to alter cell cycle parameters and subsequent analysis of the effects on neuronal differentiation (Box 1). The unifying result is that manipulations that prolong the G1 phase of precursors lead to increased neurogenic divisions and premature differentiation, whereas a shortening of G1 favours proliferative divisions and precursor expansion. It should be noted that experiments using *in utero* electroporation create transient transfection effects due to the short half-life of cyclin/cdks and dilution of plasmids through cell division. Therefore, the manipulated precursor pool then undergoes physiological differentiation 48–72 h later, and a transient shortening of G1 that expands the precursor pool then generates an excess of late-born neurons [22].

The precise mechanism behind the importance of the G1 phase in controlling neurogenesis has yet to be determined, but several hypotheses can be put forward by considering the events and molecular changes during G1, as discussed below.

Firstly, recent work identified G1 as a time of early lineage specification in human ESCs (hESCs). Endodermal specification in response to extrinsic Activin/Nodal signalling occurs only during early G1, and cells become refractory in late G1, instead adopting an alternative neuroectodermal cell fate. Mechanistically, the accumulation of active cyclin-D-cdk4/6 complexes during G1 phase results in inhibitory phosphorylation of smad2 and smad3, preventing the cellular response downstream of Activin/Nodal signalling [7]. Other direct targets of cyclin/cdks may also have key roles in precursor maintenance and neuronal differentiation (see below).

Secondly, the responsiveness of the cell during G1 may reflect the complement of transcription factors expressed at that time. Pluripotent stem cells express several key developmental regulators with a cell cycle bias. For example, FoxA2, GATA4, and Pax7 are upregulated during the G1 phase and downregulated as cells transit into S phase; therefore, G1 may represent a time when cells are lineage primed [23]. Similarly, there is evidence to suggest that basic helix-loop-helix (bHLH) proneural proteins, such as Neurogenin 2 (Ngn2) and Achaete-Scute Homologue 1 (Ascl1), which are master regulators of the neurogenic machinery (see below), adopt a cell cycle-dependent expression pattern, specifically during mid-corticogenesis (E15.5) in the mouse. Ngn2 is expressed in the late G1 phase nuclei located in the central VZ region and is excluded from the G2/M phase nuclei. By contrast, Ascl1 accumulates in early G1 nuclei. Given that Ngn2 is critical to specification of cortical neuron fate, the longer G1 phase may allow a greater accumulation of Ngn2 protein [24].

Finally, the susceptibility to extrinsic fate determinants during G1 may reflect a more permissive chromatin state. Global epigenetic changes occur in pluripotent stem cells in a cell cycle-dependent manner and this may regulate gene expression to allow a cell to respond specifically during a given cell cycle phase [23].

It is likely that multiple mechanisms operate to coordinate cell cycle, cell fate, and overt differentiation, and these may have variable importance in different cell types. For example, two populations of cortical precursor cells exit the cell cycle on E14 in the mouse, and either rapidly (Q-fast) or slowly (Q-slow) leave the VZ; fate choice of the former may be predominantly determined by cell intrinsic mechanisms, whereas the latter are influenced more by extrinsic signals [25]. Furthermore, recent work in developing chick spinal cord suggests that a shortened G2 phase in spinal precursors undergoing neurogenic divisions may be important to limit the receptive window for pro-proliferative cues from the Notch and Wnt signalling paths [26].

## Cell cycle-dependent post-translational modifications

bHLH transcription factors have key roles at multiple points during neurogenesis in the CNS, binding DNA as active heterodimers with ubiquitously expressed E proteins. Indeed, bHLH proneural determination factors, such as Ngn2 and Ascl1, are considered master regulators of neurogenesis, activating a plethora of differentiation genes that coordinate neural commitment, subtype specification, and neuronal maturation [27]. However, these factors are also instrumental in activating expression of the Notch ligand, Delta, and subsequent maintenance of the progenitor phenotype in neighbouring cells via lateral inhibition. Early work established that, at least in some cases, progenitor-associated genes have a more open chromatin state, whereas differentiation-associated genes require additional epigenetic remodelling before activation [28].

Recently, a mechanism has been described that directly links cell cycle progression in neural precursor cells with their propensity to undergo differentiation, through post-translational modification of Ngn2 [29]. These findings have allowed the development of a detailed model, whereby cdk-dependent phosphorylation of this key regulator coordinates the cell cycle control of precursor maintenance versus differentiation.

Ngn2 can be phosphorylated on up to nine serine residues, found within serine–proline (SP) pairs, and phosphorylation of these multiple sites is dependent on both the level and duration of exposure to cdk activity. Therefore, a functional response to these phosphorylation events gives a rheostat-like response to changes in cyclin-cdk activity during the cell cycle and development [29]. Indeed, when the cell cycle is active and cyclin-cdk levels are high, Ngn2 is in a (hyper)-phosphorylated form and has a reduced DNA binding affinity that is sufficient only to activate the progenitor-associated target promoters that have open chromatin. As the cell cycle lengthens, cyclin-cdk activity is reduced and Ngn2 phosphorylation decreases, resulting in an increase in DNA-binding affinity. This longer promoter dwell time by hypophosphorylated Ngn2 appears to be necessary to bring about the epigenetic remodelling and activation of downstream target promoters that drive neuronal differentiation. Thus, as cdk levels decrease, the level of progenitor gene expression remains fairly static and the expression of differentiation genes relatively increases to tip the balance in favour of differentiation [30]. Experimentally, a phosphomutant form of Ngn2 that has all nine SP sites mutated to serine–alanine (SA) and so cannot be phosphorylated by cdks, shows a significantly enhanced ability to drive neuronal differentiation both *in vitro* and *in vivo*, supporting the model presented above [29].

Finally, Ngn2 undergoes both canonical and noncanonical ubiquitination, which contribute to rapid protein turnover via the proteasome. Ngn2 displays changes in stability at different cell cycle phases, and noncanonical ubiquitination via cysteine residues may contribute to the greater turnover observed during mitosis [31]. Moreover, the *Xenopus* cdk inhibitor p27Xic1 directly stabilises the Ngn2 protein independently of its ability to regulate the cell cycle [32], again demonstrating direct links between the cell cycle machinery and post-translational control of Ngn2 protein function.

Another key proneural protein, Ascl1, also contains multiple serine/threonine–proline pairs either side of the bHLH domain. Early evidence indicates that preventing phosphorylation on these sites by mutation leads to an enhanced ability of Ascl1 to drive neuronal differentiation and maturation in both the developing *Xenopus* embryo and when used in transcription factor cocktails to reprogram human fibroblasts into neurons [33]. As with phosphomutant Ngn2 and unlike the wild type proneural proteins, phosphomutant Ascl1 is not inhibited by increased levels of cdk activity (A.P., Development, in press). Further analysis of phosphoregulation of other bHLH proneural proteins in our lab (A.P., 2014, unpublished) leads us to conclude that multisite phosphorylation either side of the bHLH domain may be a widespread mechanism to regulate proneural protein activity in response to the kinase environment.

## Proteins with dual function in cell cycle and differentiation

Tissue- and/or stage-specific expression profiles of key components of the cell cycle machinery may indicate additional, possibly context-dependent, roles during determination or differentiation (e.g., D-type cyclins, see below), beyond known roles in speeding up, slowing down, or changing the structure of the cell cycle (Table 1). Similarly, transcription factors such as the proneural proteins, which have traditionally been associated with driving differentiation, are increasingly found to influence cell cycle dynamics [10,34]. In some dual function molecules, such as some cdk inhibitors (cdkis) and Geminin, cell cycle and differentiation functions are mechanistically independent, and relate to structurally distinct regions of the protein [32,35]. In others, the interdependence between the many functions remains to be determined. However, it is increasingly clear that there are multiple bidirectional links between components of the cell cycle and differentiation machinery, some examples of which we highlight below and in Box 2.

### Cell cycle components directly influence neuronal differentiation

D-type cyclins are perhaps best known for their role in regulation of the G1 phase, activating cdk4 and cdk6 proteins to promote passage through the restriction point and commitment to cell division (Figure 1). Cdk-dependent functions in early lineage specification during G1 were discussed above [7], but an increasing array of cdk-independent functions are also being appreciated.

Despite their functional redundancy during cell cycle regulation, early functional differences between cyclin-D1 and cyclin-D2 have been described, with a specific requirement for cyclin-D2 during expansion of the BP population in the embryonic cortex. This cannot be compensated by cyclin-D1, suggesting that cyclin-D2 contributes to the evolutionary development of the enlarged supragranular layer of neurons in the primate cortex [36]. Similarly, cyclin-D2 is required for precursor maintenance in the cerebellum to ensure late-born interneurons can be generated postnatally [37].

By contrast, cyclin-D1 appears to have more proliferation-independent functions during neuronal determination and differentiation. In spinal cord, cyclin-D1 has a positive regulatory role in motor neuron differentiation, and enforced expression of cyclin-D1 in glial-restricted precursors is sufficient to confer a neurogenic capacity on these cells. However, cyclin-D2 exerts opposing effects on neurogenesis and this is attributed to differential upregulation of Hes genes; cyclin-D1 promoting Hes-6, and cyclin-D2 promoting anti-neurogenic Hes-5 [38].

Additional insights into cdk-independent gene regulation by cyclins have uncovered a direct transcriptional role of cyclin-D1 in the developing retina. Although cyclin-D1 can both activate and repress gene expression, the phenotype observed in knockout mice results from downregulated Notch1 expression; cyclin-D1 recruits activating CBP histone acetyltransferase to the Notch1 upstream regulatory region in a cdk-independent manner [39].

Expression patterns of cyclin-E also indicate a selective high-level retention in the adult murine brain, where cyclin-E has a cell cycle-independent and rate-limiting function in terminally differentiated neurons. In contrast to the usual cdk-activating role, cyclin-E sequesters cdk5 in a catalytically inactive complex, enabling the correct formation and function of synapses; however, how this correlates with other cdk5 functions in synaptogenesis remains to be determined [40].

The developing *Xenopus* embryo has a single cdk inhibitor, p27Xic1, which functions during the neuronal commitment stage and is necessary for primary neurogenesis, independent of cdk2 inhibition [32]. Subsequent studies in the mammalian cortex confirm that the N terminus of the mammalian homologue p27Kip1 confers stability to proneural protein Ngn2 and promotes neuronal differentiation, whereas the C-terminal domain is additionally able to promote neuronal migration through inhibition of RhoA signalling [35]. The Kip/Cip family of cdkis also includes p57Kip2, which similarly functions as a modular protein to regulate cortical precursor proliferation and differentiation [41], and additional cdk-independent pro-migratory functions reside in the N terminus of p57Kip2 [42].

### Proneural proteins directly influence the cell cycle

Not unexpectedly, cell cycle components are a key group of genes differentially downregulated during differentiation of murine neural stem cells [43], and transcription factors with known roles driving neuronal differentiation also have direct effects on cell cycle components. For example, overexpression of Ngn2 in mouse spinal cord precursors promotes cell cycle exit by rapidly downregulating a subset of cyclins that act at the G1–S phase transition of the cell cycle. Although gene repression is likely to be indirect, effects are evident within 6 h of overexpression, and cells are retained in G1 phase before changes in the levels of cdks or cdkis [10].

Although anti-proliferative roles for these proneural bHLH transcription factors have been long described [44], an unexpected and additional pro-proliferative role was recently revealed for Ascl1, following combined chromatin binding and genome-wide expression profiling in mouse ventral telencephalon precursor cells [34]. A key finding of this study is that endogenous Ascl1 directly binds and activates the promoters of cell cycle progression genes, such as Skp2 and E2F1, and *in vitro* studies confirm that Ascl1 functionally regulates cell cycle proliferation genes in cycling BP cells. However, overexpression of Ascl1 that concurrently induces neuronal differentiation, leads to upregulation of cell cycle-arrest genes. Thus, opposing sets of target genes display temporally distinct activation patterns. The mechanism of differential regulation has yet to be clearly elucidated, but may involve coregulation with Notch signalling that is active in precursor cells [34] or post-translational regulation of Ascl1, analogous to that described above for Ngn2 [29].

Alternatively, it may be the pattern or mode of proneural protein expression that determines the target genes activated and the balance between proliferation and differentiation [45]. The traditional view of neural progenitor maintenance via lateral inhibition was based on a ‘salt and pepper’ distribution model: differentiating neurons express proneural proteins and Delta ligand, thus activating Notch signalling and Hes genes in neighbouring cells, preventing that neighbour from similarly upregulating proneural proteins (recently reviewed in [46]). However, time-lapse imaging has since revealed a more dynamic picture, with Hes1 expression oscillating with a period of 2–3 h in neural precursor cells, and Ngn2 and Delta mRNA oscillating in antiphase due to inhibition from Hes1. This pattern changes in differentiating neurons where proneural expression becomes sustained and Hes1 is repressed, although the precise mechanism for permanent repression of Hes1 is not yet clear [47]. Furthermore, enforcing persistent Hes1 expression in precursor cells induces ectopic neuronal differentiation of neighbouring cells, indicating that it is the oscillatory nature of both proneural and Hes expression that is required for mutual activation of Notch signalling and maintenance of the precursor pool [47]. Reflecting back to the epigenetic status of different sets of target genes, oscillatory expression of proneural proteins may be sufficient to activate progenitor-associated genes with open chromatin states, whereas a more sustained expression may be required for differentiation genes [29].

Extending this theory, oscillatory expression is not confined to Ngn2. In ventral telencephalon neural precursor cells, multipotency is characterised by oscillating neurogenic and gliogenic determination factors, whereas commitment to a neuronal, oligodendrocyte, or astrocyte cell fate is associated with sustained expression of a single factor, namely Ascl1, Olig2, or Hes1, respectively. Using a new light-induced expression system with Ascl1-null cells, introduction of Ascl1 oscillations with 3-h periodicity was shown to enhance cell proliferation, whereas sustained expression was required for differentiation [45]. It will now be important to determine the mechanisms mediating the change in target gene expression patterns and, thus, governing the switch from proliferation to differentiation.

## Epigenetic mechanisms

An appreciation of context-dependent function is increasingly apparent for the temporal and spatial precision of transcription factor activity. This is likely to involve interaction with restricted cofactors, and may also be influenced by differential epigenetic landscapes. Several examples have already been presented where epigenetics can influence the balance between proliferation and differentiation: for example, G1 phase may represent a time when the cell is poised to respond to extrinsic cues due to a permissive chromatin state [23], and temporal changes in proneural target gene activation may reflect differences in the epigenetic landscape of promoters [29]. Bivalent combinations of activating (H3K4 trimethylation) and repressive (H3K27 trimethylation) histone modifications can mark developmental genes in a poised but still repressed state [48,49], and the cell cycle-associated protein Geminin appears to have an active role in maintaining this [50].

DNA methylation and histone modifications can also contribute to terminal fate restriction and long-term repression of early developmental genes. Failure to erase such marks can present a practical barrier for cellular reprogramming strategies that convert terminally differentiated cells back into a less differentiated state [48]. Further epigenetically regulated mechanisms may also be at play, illustrated by the temporal switch from neurogenesis to astrogliogenesis, which is assisted by demethylation of astrocytic genes that enable the cell to respond to activation of the JAK-STAT pathway [51]. Although miRNAs are well-established regulators in neural development [49,52], connections to the cell cycle remain poorly understood; this is likely to be a field of expanding interest, and we highlight some recent insights in Box 3.

## Concluding remarks

**Figure 2:**
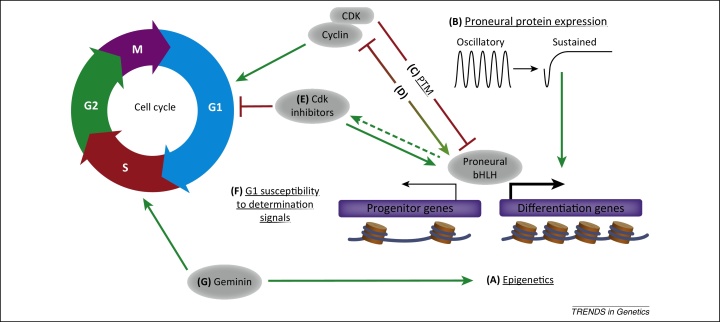
Multiple mechanisms coordinate the cell cycle and neuronal differentiation. **(A)** Proneural basic helix-loop-helix (bHLH) transcription factors have multiple direct downstream targets genes that are involved in both progenitor maintenance and in driving neuronal differentiation [34]. Progenitor-associated genes often have a relatively more accessible and open chromatin state, whereas differentiation gene promoters may require extensive remodelling [28]. The influence of the epigenetic landscape is a new and developing field of interest. **(B)** The expression pattern of proneural proteins changes during differentiation, and an oscillatory pattern is associated with the progenitor state, whereas sustained expression is required to promote differentiation [45]. **(C)** Active cyclin-cdk complexes drive progression through the cell cycle, but additionally inhibit the expression of differentiation-associated genes by post-translational modification (PTM) of proneural proteins [29,30]. **(D)** Different proneural proteins can influence cyclin-cdk complexes at a transcriptional level, either promoting cell cycle exit [10] or having both positive and negative effects depending on cell context [34]. **(E)** Cdk inhibitors promote lengthening of G1 phase, but additionally have cell cycle-independent roles to promote the activity of proneural proteins and later neuronal maturation [42,35]. Cdk inhibitors are also upregulated downstream of proneural proteins [44], but this may not be a direct regulation [10,34], indicated by the dashed line. **(F)** Lengthening of G1 phase extends the period of time that the cell is able to respond to fate-determining signals [19]. **(G)** Other components of the cell cycle machinery, such as Geminin, independently influence both the cell cycle and differentiation processes through physically separate domains of the protein [50,62–64].

The intricate balance between proliferation and differentiation is of fundamental importance in development, and we have focused on the nervous system to illustrate the multiple levels of interactions that occur to coordinate these two processes (Figure 2). Links at a transcriptional level are clear, from proneural proteins driving the expression of both cell cycle and differentiation components, to novel roles for cyclins in activating transcriptional cascades in distinct developmental contexts. Interactions at a post-translational level are also emerging as a key theme, from the dual but independent function of specific proteins in proliferation control and differentiation, to cell cycle-dependent modifications of proneural proteins that influence the nature of downstream target genes activated. In this respect, cohorts of genes can be coordinately regulated, with expression additionally influenced by chromatin; a parameter that can also be cell cycle regulated. Further work is required to elucidate the nature and associated mediators of changes in the epigenetic landscape, but this may contribute to our understanding of tissue- or stage-specific gene expression profiles.

Future studies may include a greater characterisation of cell cycle-regulated post-translational modifications of key differentiation factors, coupled with genome-wide analysis of transcription factor activity in proliferating and differentiating cells. These are likely to reveal the mechanistic basis behind at least some of the many interactions between the cell cycle and differentiation machinery, and they may also explain further the context-dependent activity of key regulators, such as the proneural proteins. Such insights will surely have far-reaching implications in our understanding of the developing nervous system, in treatment of neurological disorders and cancers, and in advancing our ability to use regenerative medicine to replace lost neurons in conditions such as stroke and spinal cord injury.

## Figures and Tables

**Table 1:** Cell cycle components directly influencing neurogenesis

Protein	Traditional cell cycle role	Role in neurogenesis	Refs
Cyclin-D1	Activator of cdk4/6 in G1 phase	Promotes differentiation of motor neurons in spinal cord	[38]
		Direct activation of Notch1 expression via CBP histone acetyltransferase recruitment	[39]
Cyclin-D2	Activator of cdk4/6 in G1 phase	Proliferation-associated role in BP cells in embryonic cortex	[36]
		Proliferation-associated role in formation of cerebellar interneurons	[37]
Cyclin-E	Activator of cdk2 in late G1 into S phase	Sequesters cdk5 to enable correct formation of synapses	[40]
p27Xic1	Cdk inhibitor	Cell fate specification in *Xenopus* retina, promoting Muller glial cells	[57]
		Required for primary neurogenesis in *Xenopus*	[32]
p27Kip1	Cdk inhibitor	Forms a repressor complex on the Sox2 promoter to inhibit expression of this progenitor-associated gene	[58]
		Promotes neuronal migration	[35]
p57Kip2	Cdk inhibitor	Promotes neuronal migration	[42]
p21Cip1	Cdk inhibitor	Required for onset of oligodendrocyte differentiation	[59]
Retinoblastoma protein	Inhibitor of G1 phase restriction point	Binds and promotes activity of NeuroD1 in pituitary	[60]
		Promotes migration of a subgroup of ventral forebrain interneurons	[61]
Geminin	Ensures DNA is replicated only once during S phase	Favours neural fate specification but then maintains progenitor state and inhibits proneural gene function	[50,62–64]

## Box 1 Manipulation of G1 and effects on neuronal differentiation

### Pharmacological inhibition of cdks

Early work demonstrated that the cdk inhibitor Olomoucine both lengthens G1 and induces a premature switch from proliferative to neurogenic precursor divisions [19]. Similarly, treatment of adult precursor cells *in vitro* with a cell permeable cdk4 inhibitor induces an increase in the percentage of cells in G1, and promotes neuronal differentiation under both self-renewing and induced differentiation culture conditions [16].

### Cdk/cyclin null phenotypes

Cyclin-D2 knockout mice show a specific defect in BP proliferation, with a substantial lengthening of G1 and premature terminal differentiation that results in microcephaly [36]. Recent work has created cdk2 and cdk4 double knockout (DKO) mice, also showing a striking reduction in cortical neurons, although DKO cells demonstrate no defects in proliferation *in vitro* due to compensatory function of cdk1 and upregulation of cyclin-D1 and cdk6. Microcephaly occurs due to a significantly increased G1 length and premature neurogenic divisions of BP cells that deplete the precursor pool and reduce long-term neuronal output [54].

### Overexpression of cyclin-cdks

*In utero* electroporation of cyclin-E1 or cyclin-D1 at E14.5 reduces G1 length and markedly expands the BP population; rates of cell cycle re-entry in BP cells are increased 80% compared with AP [55]. This differential effect is also seen with acute overexpression of cyclin-D1/cdk4 at E13.5, resulting in a 40% increase in BP cells that undergo proliferative rather than neurogenic divisions, whereas the AP population is unchanged [22].

Similar results are seen in the adult dentate gyrus. Acute overexpression of cyclin-D/cdk4 in the 6–10-week-old hippocampus cell autonomously increases the expansion of the precursor pool by increasing proliferative divisions at the expense of neurogenic divisions. When overexpression is stopped, physiological differentiation resumes and the neuronal output of the manipulated pool of precursors can be doubled. In both developing and adult brains, it is the cells with the relatively longer G1 phase that are preferentially affected by overexpression of cyclin-cdk complexes, suggesting that it is the relative change in G1 length, rather than the absolute duration, that is important [56].

## Box 2 Geminin is a dual function protein

Geminin is a dual function protein that utilises separable structural domains for independent functions. The C-terminal domain is required for cell cycle regulation, where it controls the fidelity of DNA replication and ensures that DNA is replicated just once per S phase [65]. By contrast, overexpression of the N terminus alone can induce formation of neural tissue in developing *Xenopus* embryos [62].

More recent work has established a conserved role for Geminin in initial neuronal fate specification and subsequent maintenance of the neural precursor state, preventing premature differentiation. Phenotypically, studies in mammalian cells have produced mixed results, suggesting that Geminin has context-dependent functions, or alternatively, disparities may be due to the exons deleted in the respective knockout mouse models. For example, conditional deletion of Geminin exons 5, 6, and 7 produces no neurological defects *in vivo* [66]. Yet, conditional deletion of exons 3 and 4 increases the number of AP in the rostral cortex, with a lengthening of the S phase and an increase in proliferative divisions and an associated decrease in early-born cortical neurons [14].

Mechanistically, Geminin can influence gene expression through interaction with several different transcription factors and chromatin remodelling complexes, and these interactions may vary with experimental organism or developmental stage. In gastrula-stage *Xenopus* embryos, Geminin influences neural fate by antagonising transcriptional responses to mesodermal, endodermal, and non-neural ectodermal signals. This involves functional cooperativity between Geminin and Polycomb proteins to establish a repressive chromatin state that prevents responses to subthreshold lineage specification signals [63]. Later in development, Geminin prevents premature neuronal differentiation through indirect inhibition of proneural gene function. By binding to Brg1, the catalytic subunit of a SWI/SNF chromatin-remodelling complex, Geminin inhibits Brg1 recruitment to proneural target genes [64]. Additionally, in both *Xenopus* and mammalian cells, Geminin can maintain the neural precursor state while leaving cells poised to differentiate by maintaining a bivalent epigenetic state at neural gene promoters, with both activating and repressive chromatin marks [50].

## Box 3 Recent advances in understanding epigenetics during differentiation

Epigenetic mechanisms are also influential for balancing proliferation and differentiation, but a detailed understanding of chromatin regulation during neurogenesis is lacking. Advances are being made, for example, in characterising changes in histone modifications and roles of noncoding RNAs. Here, we present a few recent examples where future research may be directed.

Firstly, miRNAs are a class of noncoding RNAs that can induce transcriptional repression of genes by complementarily binding to target mRNAs. Proliferating precursors express only a small number of miRNAs and the transition to neuronal differentiation is associated with marked upregulation of several miRNAs (such as miR-9/9*) that repress transcription factors with roles in stem cell maintenance [67]. Consistent with the role of miRNAs in corticogenesis, conditional deletion of Dicer, a component of the canonical miRNA processing path, results in disordered cell cycle kinetics, neuronal differentiation and migration, and precocious astrogenesis [68]. Mechanisms linking the cell cycle and miRNAs are also emerging; the pro-proliferative transcription factor E2F1 additionally represses transcription of a range of miRNAs that become transiently upregulated during early neuronal differentiation. Once upregulated, these miRNAs can in turn repress E2F1 transcription, promoting the transition from proliferation to differentiation [67].

Secondly, comprehensive gene expression profiles from both precursor cells undergoing proliferative and neurogenic divisions and postmitotic neurons have identified a pool of ‘switch gene’ transcripts that are unique to the neurogenic precursors. Although many are novel and previously uncharacterised, several are implicated in chromatin remodelling, or form genetic long noncoding RNAs (lncRNAs) that overlap the protein-coding regions of genes with established functions in corticogenesis. Furthermore, overexpression of individual candidate lncRNAs can produce phenotypic effects on neurogenesis, suggesting an influential role during cell fate determination [69].

Thirdly, histone modifications can influence gene expression positively and negatively. In the pre-neurogenic cortex, histone arginine methyltransferase PRMT5 is the catalytic component of an epigenetic modifier that characterises the undifferentiated state and promotes proliferation. However, postmitotic neurons express the alternative PRMT1 that modifies the same histone residue as PRMT5, but results in transcriptional activation. Furthermore, commitment to neurogenic divisions is associated with expression of Tis21 (see above), which stimulates PRMT1 activity; these sequential changes in epigenetic modifications may be intimately linked to the cell cycle and differentiation [70].

## Glossary

Apical progenitor cells (AP): Pax-6+-expressing precursor cells replicating within the ventricular zone (VZ) of the neural plate (e.g., neuroepithelial cells and radial glial cells in the cortex). Cells span the apicobasal axis and undergo mitosis at the apical (ventricular) surface.
Asymmetric self-renewing/asymmetric neurogenic division: generation of one daughter cell that continues to divide, and one more differentiated cell.
Basal progenitor cells (BP): Tbr2+-expressing precursor cells derived from apical progenitors. Cells migrate radially from the VZ into the subventricular zone (SVZ) and may undergo a limited number of asymmetric self-renewing divisions before terminal symmetric neurogenic differentiation. Sometimes also referred to as ‘intermediate progenitors’.
Embryonic stem cell (ESC): derived from blastocyst-stage embryos and capable of both long-term self-renewal and multipotent potential for daughter cells to differentiate into any of the embryonic cell lineages.
Gyrencephaly: the folding of the cerebral cortex as found, for example, in human and nonhuman primates.
Lateral inhibition: inhibitory cell–cell communication whereby a cell committed to a neural fate prevents its neighbours from adopting the same fate, maintaining them in the progenitor state via activation of Notch signalling.
Neural precursor cells (NPC): collective term for neural stem and progenitor cells.
Neural progenitor cell: a cell restricted to the neural lineage that may have more limited replicative potential before generating terminally differentiated neurons.
Neural stem cell (NSC): a somatic stem cell restricted to the neural lineage but capable of long-term self-renewal (through either symmetric proliferative or asymmetric self-renewing divisions).
Symmetric neurogenic division: generation of two daughter cells that become terminally differentiated postmitotic neurons, thus depleting the precursor pool.
Symmetric proliferative division: generation of two identical proliferating daughter cells that expand the precursor pool.

## References

[1] Zhong W., Chia W. (2008). Neurogenesis and asymmetric cell division. Curr. Opin. Neurobiol..

[2] Desai A.R., McConnell S.K. (2000). Progressive restriction in fate potential by neural progenitors during cerebral cortical development. Development.

[3] Hatakeyama J. (2004). Hes genes regulate size, shape and histogenesis of the nervous system by control of the timing of neural stem cell differentiation. Development.

[4] Nomura T. (2013). Changes in the regulation of cortical neurogenesis contribute to encephalization during amniote brain evolution. Nat. Commun..

[5] Borrell V., Reillo I. (2012). Emerging roles of neural stem cells in cerebral cortex development and evolution. Dev. Neurobiol..

[6] Betizeau M. (2013). Precursor diversity and complexity of lineage relationships in the outer subventricular zone of the primate. Neuron.

[7] Pauklin S., Vallier L. (2013). The cell-cycle state of stem cells determines cell fate propensity. Cell.

[8] McConnell S.K., Kaznowski C.E. (1991). Cell cycle dependence of laminar determination in developing neocortex. Science.

[9] Lobjois V. (2008). Forcing neural progenitor cells to cycle is insufficient to alter cell-fate decision and timing of neuronal differentiation in the spinal cord. Neural Dev..

[10] Lacomme M. (2012). NEUROG2 drives cell cycle exit of neuronal precursors by specifically repressing a subset of cyclins acting at the G1 and S phases of the cell cycle. Mol. Cell. Biol..

[11] Takahashi T. (1995). The cell cycle of the pseudostratified ventricular epithelium of the embryonic murine cerebral wall. J. Neurosci..

[12] Calegari F. (2005). Selective lengthening of the cell cycle in the neurogenic subpopulation of neural progenitor cells during mouse brain development. J. Neurosci..

[13] Arai Y. (2011). Neural stem and progenitor cells shorten S-phase on commitment to neuron production. Nat. Commun..

[14] Spella M. (2011). Geminin regulates cortical progenitor proliferation and differentiation. Stem Cells.

[15] Sakaue-Sawano A. (2008). Visualizing spatiotemporal dynamics of multicellular cell-cycle progression. Cell.

[16] Roccio M. (2013). Predicting stem cell fate changes by differential cell cycle progression patterns. Development.

[17] Coronado D. (2013). A short G1 phase is an intrinsic determinant of naïve embryonic stem cell pluripotency. Stem Cell Res..

[18] Calder A. (2013). Lengthened G1 phase indicates differentiation status in human embryonic stem cells. Stem Cells Dev..

[19] Calegari F., Huttner W.B. (2003). An inhibition of cyclin-dependent kinases that lengthens, but does not arrest, neuroepithelial cell cycle induces premature neurogenesis. J. Cell Sci..

[20] Lange C., Calegari F. (2010). Cdks and cyclins link G1 length and differentiation of embryonic, neural and hematopoietic stem cells. Cell Cycle.

[21] Sela Y. (2012). Human embryonic stem cells exhibit increased propensity to differentiate during the G1 phase prior to phosphorylation of retinoblastoma protein. Stem Cells.

[22] Lange C. (2009). Cdk4/cyclinD1 overexpression in neural stem cells shortens G1, delays neurogenesis, and promotes the generation and expansion of basal progenitors. Cell Stem Cell.

[23] Singh A.M. (2013). Cell-cycle control of developmentally regulated transcription factors accounts for heterogeneity in human pluripotent cells. Stem Cell Rep..

[24] Britz O. (2006). A role for proneural genes in the maturation of cortical progenitor cells. Cereb. Cortex.

[25] Goto T. (2004). Altered patterns of neuron production in the p27 knockout mouse. Dev. Neurosci..

[26] Peco E. (2012). The CDC25B phosphatase shortens the G2 phase of neural progenitors and promotes efficient neuron production. Development.

[27] Wilkinson G. (2013). Proneural genes in neocortical development. Neuroscience.

[28] Koyano-nakagawa N. (1999). Activation of *Xenopus* genes required for lateral inhibition and neuronal differentiation during primary neurogenesis. Mol. Cell. Neurosci..

[29] Ali F. (2011). Cell cycle-regulated multi-site phosphorylation of Neurogenin 2 coordinates cell cycling with differentiation during neurogenesis. Development.

[30] Hindley C. (2012). Post-translational modification of Ngn2 differentially affects transcription of distinct targets to regulate the balance between progenitor maintenance and differentiation. Development.

[31] Vosper J.M.D. (2009). Ubiquitylation on canonical and non-canonical sites targets the transcription factor neurogenin for ubiquitin-mediated proteolysis. J. Biol. Chem..

[32] Vernon A.E. (2003). The cdk inhibitor p27Xic1 is required for differentiation of primary neurones in *Xenopus*. Development.

[33] Pang Z.P. (2011). Induction of human neuronal cells by defined transcription factors. Nature.

[34] Castro D.S. (2011). A novel function of the proneural factor Ascl1 in progenitor proliferation identified by genome-wide characterization of its targets. Genes Dev..

[35] Nguyen L. (2006). P27Kip1 independently promotes neuronal differentiation and migration in the cerebral cortex. Genes Dev..

[36] Glickstein S.B. (2009). Cyclin D2 is critical for intermediate progenitor cell proliferation in the embryonic cortex. J. Neurosci..

[37] Leto K. (2011). Modulation of cell-cycle dynamics is required to regulate the number of cerebellar GABAergic interneurons and their rhythm of maturation. Development.

[38] Lukaszewicz A.I., Anderson D.J. (2011). Cyclin D1 promotes neurogenesis in the developing spinal cord in a cell cycle-independent manner. Proc. Natl. Acad. Sci. U.S.A..

[39] Bienvenu F. (2010). Transcriptional role of cyclin D1 in development revealed by a genetic-proteomic screen. Nature.

[40] Odajima J. (2011). Cyclin E constrains Cdk5 activity to regulate synaptic plasticity and memory formation. Dev. Cell.

[41] Mairet-Coello G. (2012). p57(KIP2) regulates radial glia and intermediate precursor cell cycle dynamics and lower layer neurogenesis in developing cerebral cortex. Development.

[42] Tury A. (2011). The cyclin-dependent kinase inhibitor p57Kip2 regulates cell cycle exit, differentiation, and migration of embryonic cerebral cortical precursors. Cereb. Cortex.

[43] Karsten S.L. (2003). Global analysis of gene expression in neural progenitors reveals specific cell-cycle, signaling, and metabolic networks. Dev. Biol..

[44] Farah M.H. (2000). Generation of neurons by transient expression of neural bHLH proteins in mammalian cells. Development.

[45] Imayoshi I. (2013). Oscillatory control of factors determining multipotency and fate in mouse neural progenitors. Science.

[46] Shimojo H. (2011). Dynamic expression of notch signaling genes in neural stem/progenitor cells. Front. Neurosci..

[47] Shimojo H. (2008). Oscillations in notch signaling regulate maintenance of neural progenitors. Neuron.

[48] Hirabayashi Y., Gotoh Y. (2010). Epigenetic control of neural precursor cell fate during development. Nat. Rev. Neurosci..

[49] Mohamed Ariff I. (2012). Epigenetic regulation of self-renewal and fate determination in neural stem cells. J. Neurosci. Res..

[50] Yellajoshyula D. (2012). Geminin regulates the transcriptional and epigenetic status of neuronal fate-promoting genes during mammalian neurogenesis. Mol. Cell. Biol..

[51] Fan G. (2005). DNA methylation controls the timing of astrogliogenesis through regulation of JAK-STAT signaling. Development.

[52] Ji F. (2013). The role of microRNAs in neural stem cells and neurogenesis. J. Genet. Genomics.

[53] Cheffer A. (2013). Cell cycle regulation during neurogenesis in the embryonic and adult brain. Stem Cell Rev..

[54] Lim S., Kaldis P. (2012). Loss of Cdk2 and Cdk4 induces a switch from proliferation to differentiation in neural stem cells. Stem Cells.

[55] Pilaz L-J. (2009). Forced G1-phase reduction alters mode of division, neuron number, and laminar phenotype in the cerebral cortex. Proc. Natl. Acad. Sci. U.S.A..

[56] Artegiani B. (2011). Overexpression of cdk4 and cyclinD1 triggers greater expansion of neural stem cells in the adult mouse brain. J. Exp. Med..

[57] Ohnuma S. (1999). p27xic1, a Cdk Inhibitor, promotes the the determination of glial cells in *Xenopus* retina. Cell.

[58] Li H. (2012). p27(Kip1) directly represses Sox2 during embryonic stem cell differentiation. Cell Stem Cell.

[59] Zezula J. (2001). p21cip1 is required for the differentiation of oligodendrocytes independently of cell cycle withdrawal. EMBO J..

[60] Batsché E. (2005). Retinoblastoma and the related pocket protein p107 act as coactivators of NeuroD1 to enhance gene transcription. J. Biol. Chem..

[61] Andrusiak M.G. (2011). Rb/E2F regulates expression of neogenin during neuronal migration. Mol. Cell. Biol..

[62] Kroll K.L. (1998). Geminin, a neuralizing molecule that demarcates the future neural plate at the onset of gastrulation. Development.

[63] Lim J-W. (2011). Geminin cooperates with Polycomb to restrain multi-lineage commitment in the early embryo. Development.

[64] Seo S. (2005). The SWI/SNF chromatin remodeling protein Brg1 is required for vertebrate neurogenesis and mediates transactivation of Ngn and NeuroD. Development.

[65] McGarry T.J., Kirschner M.W. (1998). Geminin, an inhibitor of DNA replication, is degraded during mitosis. Cell.

[66] Schultz K.M. (2011). Geminin-deficient neural stem cells exhibit normal cell division and normal neurogenesis. PLoS ONE.

[67] Palm T. (2013). A systemic transcriptome analysis reveals the regulation of neural stem cell maintenance by an E2F1-miRNA feedback loop. Nucleic Acids Res..

[68] McLoughlin H.S. (2012). Dicer is required for proliferation, viability, migration and differentiation in corticoneurogenesis. Neuroscience.

[69] Aprea J. (2013). Transcriptome sequencing during mouse brain development identifies long non-coding RNAs functionally involved in neurogenic commitment. EMBO J..

[70] Chittka A. (2012). Transcription factor positive regulatory domain 4 (PRDM4) recruits protein arginine methyltransferase 5 (PRMT5) to mediate histone arginine methylation and control neural stem cell proliferation and differentiation. J. Biol. Chem..

